# Reemergence of Human African Trypanosomiasis Caused by *Trypanosoma brucei rhodesiense*, Ethiopia

**DOI:** 10.3201/eid3001.231319

**Published:** 2024-01

**Authors:** Adugna Abera, Tihitina Mamecha, Ebise Abose, Belachew Bokicho, Agune Ashole, Tesfahun Bishaw, Abinet Mariyo, Buzayehu Bogale, Haileyesus Terefe, Henok Tadesse, Mahlet Belachew, Hailemariam Difabachew, Araya Eukubay, Solomon Kinde, Abraham Ali, Feyesa Regasa, Fikre Seife, Zeyede Kebede, Mesfin Wossen, Getachew Tollera, Mesay Hailu, Nigus Manaye, Nick Van Reet, Gerardo Priotto, Johan van Griensven, Myrthe Pareyn, Geremew Tasew

**Affiliations:** Ethiopia Public Health Institute, Bacterial, Parasitic, and Zoonotic Research Directorate, Addis Ababa, Ethiopia (A. Abera, M. Belachew, H. Difabachew, A. Eukubay, S. Kinde, A. Ali, G. Tasew);; Selamber Primary Hospital, Kucha Alfa District, Selamber, Ethiopia (T. Mamecha, A. Mariyo, B. Bogale);; Public Health Emergency Management, Ethiopia Public Health Institute, Addis Ababa (E. Abose, H. Tadesse, F. Regasa, M. Wossen);; South Nations Nationalities People’s Region Health Bureau, Hawassa, Ethiopia (B. Bokicho, A. Ashole, H. Terefe);; Federal Ministry of Health Ethiopia, Addis Ababa (T. Bishaw, F. Seife);; World Health Organization Country Office, Addis Ababa (Z. Kebede, N. Manaye);; Ethiopia Public Health Institute, Addis Ababa (G. Tollera, M. Hailu);; World Health Organization Center for Research and Training on Human African Trypanosomiasis Diagnostics, and Trypanosoma Unit, Institute of Tropical Medicine, Antwerp, Belgium (N. Van Reet);; World Health Organization, Geneva, Switzerland (G. Priotto);; Unit of Neglected Tropical Diseases, Institute of Tropical Medicine, Antwerp (J. van Griensven, M. Pareyn)

**Keywords:** human African trypanosomiasis, Trypanosoma brucei, vector-borne infections, parasites, neurologic illnesses, tsetse flies, animal reservoirs, Ethiopia

## Abstract

We report 4 cases of human African trypanosomiasis that occurred in Ethiopia in 2022, thirty years after the last previously reported case in the country. Two of 4 patients died before medicine became available. We identified the infecting parasite as *Trypanosoma brucei rhodesiense*. Those cases imply human African trypanosomiasis has reemerged.

Human African trypanosomiasis (HAT), also known as African sleeping sickness, is caused by different subspecies of the blood parasite *Trypanosoma brucei*. *T. brucei rhodesiense* mainly affects livestock and wildlife but sporadically spills over into humans, causing an acute disease that progresses quickly. Without prompt appropriate treatment, patient prognosis is poor; case-fatality rate is almost 100%. Tsetse flies (*Glossina* spp.) transmit the parasites (*1*). HAT was first reported in Ethiopia in 1967 in the Gambella region (*2*). Sporadic cases were reported in that area until 1991 (*3*). 

In 2022, four cases of HAT were reported from the Kucha Alfa district, Gamo zone, South Nations Nationalities Peoples’ Region (SNNPR), Ethiopia. Because >3 decades had passed since the last reported case in Ethiopia, no surveillance or reporting systems existed. HAT is also not included in the national tropical diseases disease roadmap list (*4*). Necessary resources for case management were lacking at the time of the outbreak. We developed a case-series report using data from patients’ hospital records to describe activities and processes used to respond to the recent cases. Each case patient provided informed written consent or assent from parents if patient was <18 years of age. 

## The Study

All 4 case-patients with diagnosed HAT experienced fever, headache, insomnia, and a reduced level of consciousness; all were from near the Omo River area in the Kucha Alfa district of Gamo Zone, SNNPR (Figure). We initially sought to rule out malaria by microscopy of blood film at Selamber Primary Hospital (Selamber, Ethiopia). For each case-patient, we performed a complete blood count and blood chemistry analysis (Table 1). Dried blood spot samples retrieved from 1 case-patient in October 2022 were sent to the Institute of Tropical Medicine in Antwerp, Belgium, where the parasite was confirmed by molecular analysis as *T. brucei rhodesiense*. 

**Figure:**
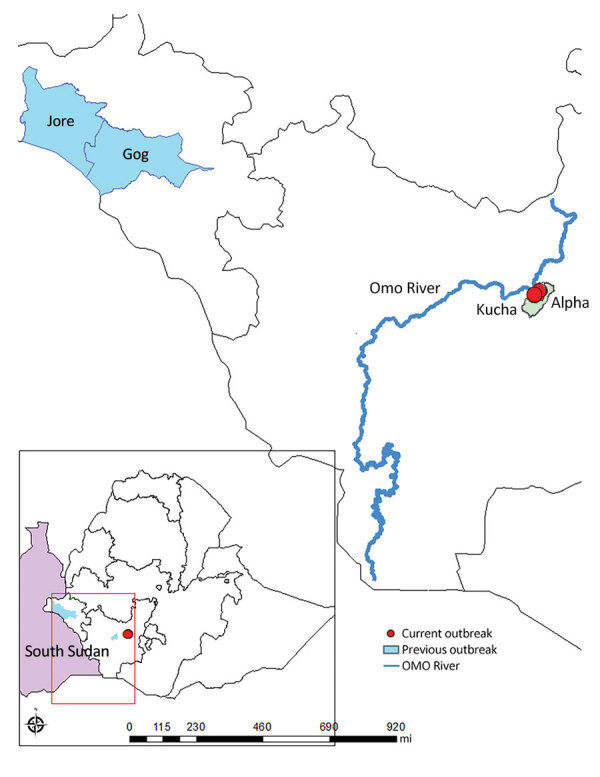
Locations of past and present human African trypanosomiasis outbreaks in Ethiopia. Areas in light blue indicate location of 1969–1970 outbreak and last case report in the Goge and Jore Districts in the Gambella region. Red dot indicates location of 2022 outbreak in Kucha Alfa District, Gamo Zone, South Nations Nationalities Peoples’ Region. Inset shows location of primary map area.

**Table 1:** Laboratory data and clinical information for 4 human African trypanosomiasis case-patients, Selamber Primary Hospital, Kucha Alfa District, South Nations Nationalities Peoples’ Region, Ethiopia, 2022*

Laboratory data	Case 1	Case 2	Case 3	Case 4
Leukocytes, × 10^9^ cells/L	4.7	5	7.5	4
Hemoglobin, g/dL	6.6	8	7.3	10
MCV, fL	88	91.3	96	89
Platelets, × 10^3^/μL	120	110	143	90
SGOT, U/L	5	3.6	83.7	21.3
SGPT, U/L	0.5	4.4	26.3	37
ALP, U/L	18.5	65	70	200
Giemsa-stained blood film	*T. brucei* spp. positive	*T. brucei* spp. positive	*T. brucei* spp. positive	*T. brucei* spp. positive
Test blood film for malaria	Yes	Yes	Yes	Yes
PCR test done	No	No	Yes	No

*ALP, alkaline phosphatase; MCV, mean corpuscular volume; SGOT, serum glutamic-oxaloacetic transaminase; SGPT, serum glutamic-pyruvic transaminase.

We reported the cases to the Ethiopia Public Health Institute after the second case. A working group from the Ethiopia Ministry of Health and World Health Organization Ethiopia country office was immediately established to make resources available for case management and case reporting. 

Fever, headaches, and joint and muscle discomfort were the most frequently expressed clinical signs and symptoms among the 4 confirmed case-patients. All manifested acute malnutrition, loss of consciousness, and insomnia. The first 2 case-patients died because appropriate treatment was unavailable in Ethiopia at that time. By the time the third case-patient sought treatment, HAT had progressed to stage 2; after performing cerebrospinal fluid analysis, we treated the patient with melarsoprol. Unfortunately, the patient developed a drug-induced encephalopathic syndrome with high-grade fever and generalized seizures and subsequently died. The fourth patient was successfully treated (Table 2). 

**Table 2:** Sociodemographic and treatment information for 4 human African trypanosomiasis case-patients, Selamber Primary Hospital, Kucha Alfa District, South Nations Nationalities Peoples’ Region, Ethiopia, 2022*

Descriptions	Case 1	Case 2	Case 3	Case 4
Age	18 mo	11 y	20 y	7 y
Sex	F	M	M	M
Date of onset	2022 Mar 20	2022 Apr 5	2022 Jul 13	2022 Sep 11
Date of diagnosis	2022 Apr 15	2022 May 28	2022 Oct 20	2022 Oct 29
CSF findings	Motile *T. brucei* spp. positive	Motile *T. brucei* spp. positive	Motile *T. brucei* spp. positive	Motile *T. brucei* spp. positive
Treatment	None	None	Melarsoprol (2.2 mg/kg/d)	Melarsoprol (2.2 mg/kg/d)
Final outcome	Died	Died	Died	Cured

*CSF, cerebrospinal fluid

Responses to the outbreak, provided with World Health Organization support, included making laboratory supplies, medicines, technical guidelines, healthcare staff training, and job aids (e.g., fact sheets, checklists, manuals) available for clinically suspected HAT, and establishment of a surveillance system. When the outbreak began, no national guidelines, training materials, or protocols for HAT existed, meaning few disease prevention and control measures were available. 

The Kucha Alfa district is surrounded by the Omo River, where tsetse flies are abundant and cattle come to drink water. Furthermore, the district is close to the Maze National Park, which is home to much wildlife and livestock. The proximity of tsetse fly habitat, animal reservoirs, and humans increases the likelihood of interaction between humans and infected wildlife and consequently the risk for infectious spillover, including from *T. brucei rhodesiense*. The areas in which the recent cases originated are rural and hard to reach, and residents have little access to medical facilities. Local persons usually first visit primary healthcare facilities in their village, then a district health center, and only after unsuccessful diagnosis or treatment do they seek care at Selamber Primary Hospital. That multistep progression substantially delays diagnosis and reduces chances for a good prognosis. Furthermore, the absence of laboratory supplies, gaps in health professionals' knowledge and expertise related to HAT, and lack of resources for active surveillance and case management further hamper timely diagnosis and treatment initiation. 

According to 1 study (*5*), animal trypanosomiasis in Ethiopia poses a serious threat to livestock and agricultural productivity. SNNPR states comprise ≈75% of the area in Ethiopia conducive to tsetse fly habitat. Most risk factors enabling the transmission of HAT are present in the Kucha Alfa district. Many locations in the Gamo and Gofa zones in SNNPR, including savannah-covered national parks, river basins, and bushy land areas, together with favorable average temperatures, support tsetse fly reproduction (*6*). 

## Conclusions

HAT has reemerged in Ethiopia in a different geographic region from where previous cases were reported 30 years earlier. Four confirmed case-patients were recently admitted to Selamber Hospital, providing evidence of ongoing transmission of the disease. Left untreated, HAT is almost always fatal, and the prognosis is generally poor even with treatment. Resources that can be established quickly and mobilized for surveillance, detection, reporting, diagnosis, and treatment of new cases are urgently needed. It is imperative to raise awareness of HAT by including it in the list of national tropical diseases in Ethiopia. Collaborative partnerships, including with One Health programs, are critical for designing control strategies, and additional areas that might be vulnerable to HAT should be mapped using the worldwide HAT atlas (*7*). 
